# Development of SSR Databases Available for Both NGS and Capillary Electrophoresis in Apple, Pear and Tea

**DOI:** 10.3390/plants10122796

**Published:** 2021-12-17

**Authors:** Sogo Nishio, Miyuki Kunihisa, Fumiya Taniguchi, Hiromi Kajiya-Kanegae, Shigeki Moriya, Yukie Takeuchi, Yutaka Sawamura

**Affiliations:** 1 Institute of Fruit Tree and Tea Science, NARO, Tsukuba 305-8605, Japan; miyuky@affrc.go.jp (M.K.); fumiya@affrc.go.jp (F.T.); takeuchiy120@affrc.go.jp (Y.T.); 2 Research Center for Agricultural Information Technology, NARO, Tokyo 105-0003, Japan; kanegaeh976@affrc.go.jp; 3 Institute of Fruit Tree and Tea Science, NARO, Morioka 020-0123, Japan; moriyas@affrc.go.jp (S.M.); ysawa@affrc.go.jp (Y.S.)

**Keywords:** variety discrimination, microsatellite, automation

## Abstract

Developing new varieties in fruit and tea breeding programs is very costly and labor-intensive. Thus, establishing a variety discrimination system is important for protecting breeders’ rights and producers’ profits. Simple sequence repeat (SSR) databases that can be utilized for both next-generation sequencing (SSR-GBS) and polymerase chain reaction–capillary electrophoresis (PCR-CE) would be very useful in variety discrimination. In the present study, SSRs with tri-, tetra- and pentanucleotide repeats were examined in apple, pear and tea. Out of 37 SSRs that showed clear results in PCR-CE, 27 were suitable for SSR-GBS. Among the remaining markers, there was allele dropout for some markers that caused differences between the results of PCR-CE and SSR-GBS. For the selected 27 markers, the alleles detected by SSR-GBS were comparable to those detected by PCR-CE. Furthermore, we developed a computational pipeline for automated genotyping using SSR-GBS by setting a value “α” for each marker, a criterion whether a genotype is homozygous or heterozygous based on allele frequency. The set of 27 markers contains 10, 8 and 9 SSRs for apple, pear and tea, respectively, that are useful for both PCR-CE and SSR-GBS and suitable for automation. The databases help researchers discriminate varieties in various ways depending on sample size, markers and methods.

## 1. Introduction

Fruit and tea breeding requires tremendous cost and time to release varieties because of their long juvenile stage and large plant size [1]. Once elite genotypes are selected and popularized by producers and consumers, the genotypes are able to be duplicated easily by clonal propagation. For this reason, it is difficult to protect breeders’ rights because breeders cannot control illegal spreads of scions. The period of protection for perennial plants is long, 25 years, as determined by the International Union for the Protection of New Varieties of Plants. Unlike vegetable and flower breeding programs, which are mainly supported by private companies, fruit and tea breeding programs have been mainly supported by governments, universities and individuals interested in plant breeding [2,3,4]. In Japan, because some elite new varieties registered by public research organizations were transported to other countries without authorization, Japanese organizations and breeders have started to register varieties in other countries and have engaged in developing variety identification methods that must be robust and reliable [5]. To further develop varieties for fruit and tea, it is important to protect breeders’ rights and continue organized breeding programs.

Simple sequence repeats (SSRs) are neutral markers that are used mainly in population genetics as well as in conservation ecology and phylogenetic analysis [6,7,8,9]. These markers have also been applied in sensitive studies such as forensic analyses [10], parentage analyses [11,12,13] and variety discrimination [14,15]. However, SSR detection has been difficult to automate because fragments from polymerase chain reaction–capillary electrophoresis (PCR-CE) have usually been evaluated by eye to determine genotypes. Due to limited multiplexing in PCR, the cost and time to determine genotypes has remained high. Slippage and stutter bands in fragment analysis have made allele determination difficult, resulting in the possibility of increased mis-genotyping [16]. Recently, more and more single-nucleotide polymorphism (SNP) analyses based on genotyping by sequencing (GBS) or double-digest restriction-site-associated DNA sequencing (ddRAD-seq) have been applied to the genotyping of plant materials [17,18]. Even so, SSRs have some advantages. First, genetic databases for SSRs were developed in many species [19,20,21,22], and some of the materials applied in the preparation of these databases were blighted and became unavailable. By using these same SSR marker sets, researchers can compare past materials with current ones. Second, if the quality and quantity of DNA are too low to construct libraries by GBS or ddRAD-seq, PCR-CE can be applied to SSRs. Therefore, SSRs can even be used to discriminate varieties in processed products or to genotype single pollen grains [23,24]. In addition, PCR-CE with SSRs can be applied easily and flexibly to plant collections of any size without utilizing next-generation sequencing (NGS).

Recently, some methods that apply NGS techniques for genotyping SSRs, called SSR-GBS [25] or SSR-seq [26], have been developed, and some of the disadvantages of SSRs have been overcome. These methods have speeded up genotyping, determined allele size precisely and identified SNPs in SSR flanking regions. However, manual scoring is still necessary to determine genotypes for some markers, especially for dinucleotide repeats with stutter bands [27]. Some software or pipelines have been developed for automated SSR allele calling [25,26,28], but no studies have tested whether the genotypes of a variety collection determined by SSR-GBS and PCR-CE are comparable for variety discrimination. Additionally, the repeatability necessary for variety discrimination has not been adequately confirmed for SSR-GBS.

To overcome these disadvantages of SSRs, databases that can be utilized for both PCR-CE and SSR-GBS would be very helpful for use in variety discrimination. Depending on the objective, researcher preference and sample size, researchers can select a suitable way to identify genotypes by using these databases. The objective of the present study was to select SSR markers that are suitable for both PCR-CE and SSR-GBS and to validate their repeatability in SSR-GBS. In order to develop automatic allele calling and to create a pipeline that is easy to implement, we mainly used tri-, tetra- and pentanucleotide repeats that do not produce stutter bands. To assess stability and repeatability, we replicated the SSR-GBS analysis in three different species: apple (*Malus domestica* Borkh.), pear (*Pyrus pyrifolia* Nakai.) and tea (*Camellia sinensis* (L.) O. Kuntze).

## 2. Results

### 2.1. Genotyping by PCR-CE

Prior to determining the SSR genotypes in each variety, SSR positions were confirmed using BLAST+ [29] against the ‘Golden Delicious’ double-haploid (GDDH13) apple genome [30], the Japanese pear ‘Nijisseiki’ genome [31] and a draft genome of *C. sinensis* var. *sinensis* ‘Shuchazao’ (CSS-SCZ) [32] (Table S1). All of the markers except Hi15h12 in apple and TM350 in tea were matched to specific mapped regions of reference genome sequences within repeat-containing regions. Hi15h12 was mapped to fictive chromosome (0) of GDDH13, and TM350 was mapped to contig805 of CSS-SCZ. The remaining markers were mapped to different chromosome regions within each genome, which is beneficial for variety discrimination.

The markers identified for each species were successfully genotyped by PCR-CE in all of the corresponding varieties (43 apple, 29 pear and 44 tea) without any missing values (Table 1 and  S2–S4). The SSR fragments were clear, and signals for each allele were constant for each marker. The average observed heterozygosity, expected heterozygosity and polymorphism information content were, respectively, 0.62, 0.59 and 0.52 in apple, 0.46, 0.41 and 0.35 in pear and 0.56, 0.52 and 0.46 in tea (Table 2). In each of the three variety collections, all of the varieties used in this study were successfully differentiated based on genotypes by using those marker sets. The unweighted pair group method with arithmetic mean (UPGMA) dendrograms were created in each variety collection, showing some cultivars that contain the genetic structure of different species are genetically distant from the main varieties used in this study (Figure S1). For example, JM varieties developed from crossing between *M. prunifolia* Borkh and *M. pumila* Miller in apple formed a subgroup which is distant from *M. domestica* varieties. ‘Cha Chukanbohon No. 3’, which is *C. sinensis* var. *assamica*, ‘Cha Chukanbohon No. 6’, which is a chance seedling of *C. taliensis* and their relatives formed a subcluster that is distant from the main tea varieties used in this study (*C. sinensis* var. *sinensis*).

### 2.2. Genotyping by SSR-GBS

Illumina short-read sequencing data from 9.5 M reads, 7.7 M reads and 7.7 M reads were obtained from the 2 replicates of 43 varieties of apple (average of 110.2 K reads), 29 varieties of pear (average of 132.9 K reads) and 44 varieties of tea (average of 133.0 K reads), respectively. After combining the reads from forward and reverse sequences, 4.2 M, 3.8 M and 3.7 M reads were obtained, respectively. The average number of reads per variety was 49.0 K reads for apple, 66.5 K for pear and 51.4 K for tea. The reads were then demultiplexed based on the primer sequences. The average number of reads per marker ranged from 14 (TM336) to 25,406 (TsuGNH194), with an average of 3866 (Table 3). All 12 SSR markers in apple had an average of more than 50 reads, as did 9 of 10 SSRs in pear and 14 of 15 SSRs in tea. Because the markers that had a small number of reads had lower reliability, they were excluded from further analyses. The numbers of reads in the replicated analysis were similar to those in the original analysis. For example, in tea, the number of reads was highest in MSE0354 (10,510 and 8783) and lowest in TM336 (14 and 12) for the 2 replicated analyses. There were several markers that had an insufficient number of reads in a specific variety even though the overall average number of reads was high. The numbers of reads obtained for TsuGNH164 in ‘Oushuu’ and CsFM1595 in ‘Cha Chukanbohon No. 6’ were quite low, making it impossible to determine the genotypes of these markers in these varieties.

**Figure 1:**
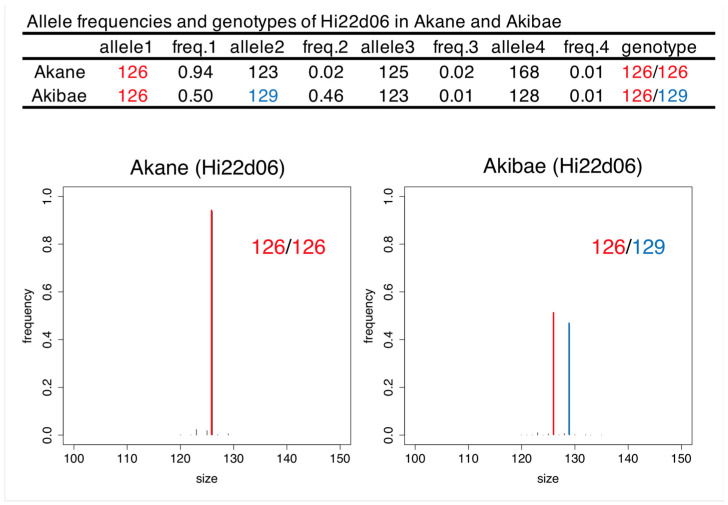
Allele frequencies, genotypes and digital fragments of Hi22d06 in ‘Akane’ and ‘Akibae’ obtained by SSR-GBS. The first alleles and second alleles were indicated in red and blue, respectively.

**Figure 2:**
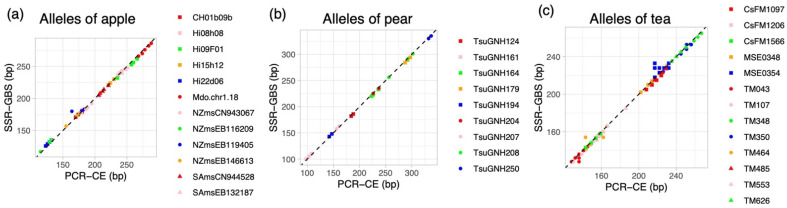
Comparisons of alleles detected by PCR-CE and SSR-GBS in apple (**a**), pear (**b**) and tea (**c**). The alleles’ length (bp) detected in capillary electrophoresis was compared against digital allele length in SSR-GBS.

Using the pipeline described in Materials and Methods, we calculated the allele frequencies for each marker in each variety. For each marker, we retrieved allele frequencies for only the four most common alleles, which provided enough information to call the genotypes and understand the details of the PCR products (Figure 1). Then, each genotype was estimated manually based on allele frequency. In most cases, the allele frequency of the first allele was >0.8 when the genotype was homozygous and around 0.4 when the genotype was heterozygous. The distribution of allele frequencies fluctuated depending on the marker owing to background arising during amplification and sequencing. For CH01b09b, which has dinucleotide repeats, genotyping errors were found in ‘Jonathan’ and ‘Sensyu’ because of the presence of stutter bands. We used tri-, tetra- and pentanucleotide repeats for all markers except for CH01b09b and Mdo.chr1.18, so there were few stutter bands for the other markers. The detected sizes of some alleles in SSR-GBS were obviously different from those detected in PCR-CE (Figure 2). In particular, NZmsEB119405 in apple, and MSE0354, TM043, TM350 and TM464 in tea, showed allele dropout (null alleles) in SSR-GBS, meaning that some alleles detected in PCR-CE were undetected in SSR-GBS (Figure 2). Except for the cases of allele dropout, the alleles detected by SSR-GBS corresponded to those detected by PCR-CE. There were some small differences between digital sizes in SSR-GBS and the artificially determined sizes in PCR-CE, which ranged from −4 bp to 3 bp, but this difference could be corrected by adding or subtracting the indicated value for each marker (Table 3). Following this analysis, 10 SSRs for apple, 8 for pear and 9 for tea were selected as suitable markers to use in SSR-GBS for variety discrimination (Tables S5–S7). All of the varieties used in this study could be successfully differentiated based on genotypes determined by those marker sets.

### 2.3. Automated Genotyping in SSR-GBS and Repeatability

To develop an accurate automated genotyping system, we set the value of a new variable, α, for each marker (Table 4). The value of α is used as a criterion whether a genotype is homozygous or heterozygous. If the first (most common) allele frequency is more than α, the genotype is assumed to be homozygous. If the first allele frequency is less than or equal to α, the genotype is called heterozygous for the first and second alleles. The range of α was largest (estimated as 0.44) for TsuGNH207 and TsuGNH250, and it was smallest for Mdo.chr1.18 (estimated as 0.01). The α for Hi15h12 showed low values (0.34–0.52). This marker produced some slippage reads, resulting in alleles from PCR-CE that were 1 bp larger than the target alleles in SSR-GBS. The α values for TsuGNH179 and TM533 were high (0.89–0.97 and 0.87–0.97, respectively). For these markers, the frequency of the second allele was sometimes less than that of the slippage and stutter band from the first allele, probably due to sequence variation in the primer binding site. Because of this fluctuation, we set the optimum α value for each marker based on the average of the minimum and maximum values, which ranged from 0.43 to 0.98 and averaged 0.75.

To estimate the repeatability of the analyses, we conducted replicated analyses with a slight change in the multiplex PCR set. In the first replicate, we used all of the primers for each species in a single multiplex PCR, whereas in the second replicate, the primers for each species were split into two sets. Otherwise, the steps were the same. The correlations of the first allele frequency among 2 replicated analyses were quite high (0.98–1.00) regardless of the marker, suggesting that this method has high repeatability when the same procedure and conditions are used.

## 3. Discussion

We developed databases suitable for both PCR-CE and SSR-GBS containing 43 varieties with 10 SSRs for apple, 29 varieties with 9 SSRs for pear, and 44 varieties with 8 SSRs for tea. We were able to distinguish all the varieties that covered most of the varieties planted in Japan; even the number of the markers were around ten. The databases were available to protect breeders’ rights of those varieties. It can also help researchers discriminate varieties in various ways depending on sample size and number of markers. The cost of SSR-GBS is lower than that of PCR-CE when the number of varieties tested is large [33]. The allele scores for PCR-CE and SSR-GBS differed somewhat (Table 3, Figure 2) because the allele sizes fluctuated depending on the type of fluorescent dyes and amplified nucleotide composition. By using the varieties in this study as controls, researchers can adjust the scores of PCR-CE to those of SSR-GBS and vice versa.

SSR-GBS has mainly been used in studies of population genetics, and few studies related to variety discrimination have been conducted. Here, we were able to select SSRs that were suitable for variety discrimination using SSR-GBS. Out of 37 SSRs tested in this study, 33 were successfully amplified (all except for TsuGNH164, TsuGNH184, CsFM1595 and TM336), but only 27 were suitable for SSR-GBS. The rate of successful PCR amplification was similar to those in other studies using SSR-GBS [25,34,35]. Out of the 27 SSRs suitable for automated genotyping, 19 had a range of α values of more than 0.2, suggesting that those markers had high reliability for SSR-GBS. Although the digital allele size determined by SSR-GBS is unambiguous, marker allele frequencies may fluctuate because of differences in enzymes, thermal cyclers and other laboratory equipment. The markers that have a greater range of α have equal amounts of amplification of the first and second alleles when heterozygous and have small numbers of nonspecific PCR products. Thus, those markers could be used as anchoring markers to connect databases. One of the merits of SSR-GBS is that it can detect length homoplasy (the occurrence of nonhomologous fragments of the same size) because it reveals SNPs [25]. However, we did not use SNPs to create the databases because the objective of this study was to develop databases available for both NGS and capillary electrophoresis that do not focus on SNPs but rather on the sizes of the PCR products.

Applying multiplex PCR and/or mixing several PCR products is necessary for NGS because the NGS platform needs sequence diversity in order to produce high-quality reads. On the other hand, applying different enzymes suitable for multiplex PCR at the same annealing temperature produced the differences between the results of SSR-GBS and PCR-CE. In this study, some allele dropouts were observed in SSR-GBS, as has been observed in other studies [33,36]. Each primer used in this study has an optimal annealing temperature, but it was not applied in these experiments, because the cost of genotyping and labor for the experimental procedure would increase if we subdivided the marker set for multiplex PCR. It is possible that some primers did not amplify alleles that had somewhat different sequences in the primer binding site. In fact, allele dropout was observed in varieties that were genetically distant from the main varieties in the database, i.e., JM varieties, which were developed from crossing between *M. prunifolia* Borkh and *M. pumila* Miller in apple; ‘Oushuu’, one of whose ancestors is Chinese pear; and ‘Cha Chukanbohon No. 3’, which is *C. sinensis* var. *assamica* introduced from India (Figure S1).

Although the accuracy of SSR-GBS, which suffers from allele dropout, is lower than PCR-CE, there are merits of SSR-GBS such as ease of automation and digital allele length, which does not vary among laboratories. We arranged the pipeline for SSR-GBS using the pipeline of Tibihika et al. [25] as a reference. Our pipeline is useful for complete automation of genotyping after the user sets the value of α for each marker. By using fastq reads, primer set sequences and α, the genotype score is easily obtained. The pipeline is simple and is composed of four files with the following functions: (1) combining the pairs of fastq files, (2) demultiplexing based on markers, (3) counting alleles and (4) determining genotypes. This pipeline works in a basic Linux system and is useful not only for variety discrimination but also for marker-assisted selection (MAS). Because the important molecular markers for fruit breeding programs were developed using SSRs and indels, the SSR-GBS pipeline can be applied to practical MAS with a slight change. Specifically, by examining four major alleles, the extent of slippage and stutter bands can be estimated because those increase the frequencies of third and fourth alleles. Even though the third and fourth alleles are not necessary for the determination of genotypes in diploid species, output of that information helps to reduce genotyping errors due to artifacts and helps to confirm whether the result of automatic genotyping is accurate.

In conclusion, the databases of apple, pear and tea developed here can provide basic information for PCR-CE and SSR-GBS. These databases and the methods developed in this study are useful for variety discrimination and protecting breeders’ rights. We also identified markers that have high reliability and are suitable for the automation of SSR-GBS. These highly reliable markers can help to connect databases and facilitate automated genotyping using a simple pipeline.

## 4. Materials and Methods

### 4.1. Plant Materials

We used a total of 116 varieties consisting of 43 varieties of apple, 29 varieties of pear and 44 varieties of tea (Table 2). The varieties of apple and pear were preserved at the Institute of Fruit Tree and Tea Science, NARO, in Morioka and Tsukuba, respectively. The tea varieties were preserved at the Institute of Fruit Tree and Tea Science, NARO, Makurazaki, Green Tea Laboratory; Saitama Prefectural Agriculture and Forestry Research Center; and Tea Branch Facility, Miyazaki Agricultural Research Institute. Genomic DNA was extracted from young leaves with a DNeasy Plant Mini Kit (Qiagen, Germany). To understand an overview of genetic relationships among varieties, UPGMA dendrograms were created using R package “pvclust” [37]. The confidence levels for branches of the dendrograms were determined by calculating approximately unbiased (AU) *p*-values using multiscale bootstrap resampling based on 10,000 replications.

### 4.2. SSR Genotyping for PCR-CE

The SSR markers used in this study are listed in Table S1 and Table 2. For apple, 12 SSRs that were mainly tri- and tetranucleotide SSRs and that showed clear fragments in PCR-CE in prior analyses were used for genotyping [22,38,39,40,41]. The 10 SSRs for pear were similar to those developed in the study by Yamamoto et al. [42], which were composed of tetra- and pentanucleotide repeats. For tea, 15 SSRs previously developed and composed of tetra- and pentanucleotide repeats were used in this experiment [43,44]. BLAST+ was used to estimate the positions of the SSRs in each reference genome [29]. The recent reference genomes were mainly composed of pseudomolecules that covered most of the genes. Chromosome positions of markers were mapped easily to design and to select optimal marker sets in genetic studies. By using reference genomes, researchers can also conduct high through-put genotyping [17,18]. PCR amplification was performed in a 10-µL solution containing 5 µL of 2× Green GoTaq G2 Hot Start Master Mix (0.4 mM each dNTP, Taq DNA polymerase and 4 mM MgCl_2_, pH 8.5; Promega, Madison, WI, USA), 20 pmol of each forward primer labeled with a fluorescent dye (5-FAM, 5-HEX, 5-VIC or 5-NES) and unlabeled reverse primer, and 10 ng of genomic DNA. Amplification was performed in 35 cycles of 94 °C for 1 min, 55 °C for 1 min, and 72 °C for 2 min. PCR products were separated and detected with a 3130xl Genetic Analyzer or a SeqStudio Genetic Analyzer (Life Technologies, Carlsbad, CA, USA). The size of each amplified band was determined manually by comparison with a set of internal-standard DNA fragments (400HD ROX, Life Technologies) in GeneMapper software v. 5.0 (Life Technologies).

### 4.3. SSR Genotyping Using SSR-GBS

**Figure 3:**
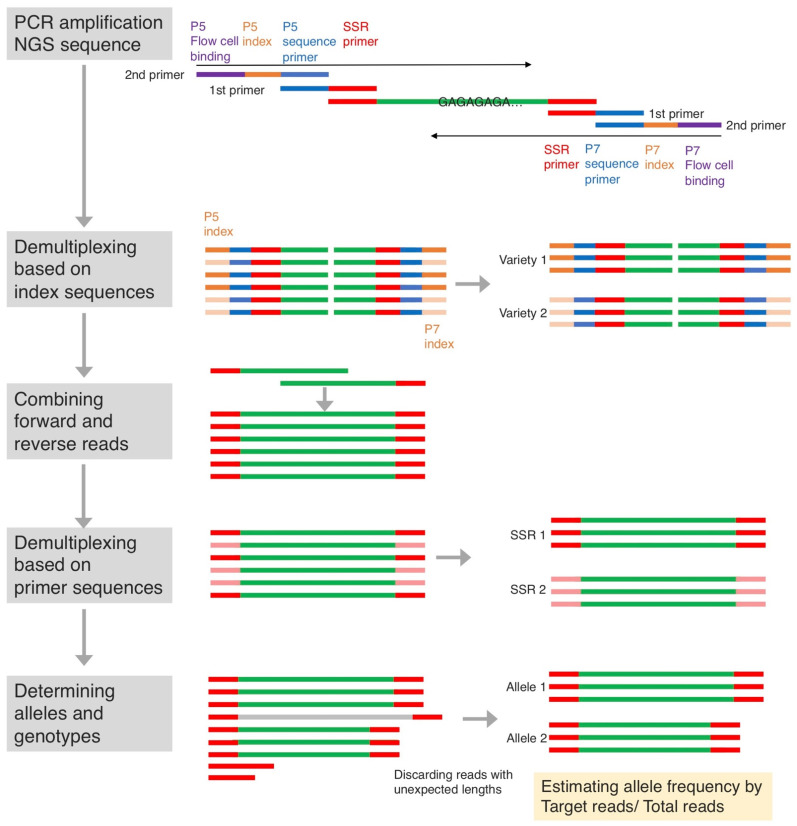
Summary of SSR-GBS procedure used in this study. Green indicates the SSR being amplified, with SSR primer annealing sequences (red) on each end. A different set of indexes (orange) is added to each sample to allow the sequences from that sample to be distinguished after multiplexing. The sequences of P5 flow cell binding, P5 sequence primer, P7 flow cell binding and P7 sequence primer were AATGATACGGCGACCACCGAGATCTACAC, ACACTCTTTCCCTACACGACG, CAAGCAGAAGACGGCATACGAGAT and GTGACTGGAGTTCAGACGTGT, respectively.

SSR-GBS was performed based on the methods described in previous reports with slight changes in sequence data analysis [25,34]. When constructing each library for SSR-GBS, two-step PCR was applied to add the Illumina adapter sequences that are required for Illumina flow-cell binding (Figure 3). The first PCR was performed using specific primers extended with the part of the Illumina adapters P5 (ACACTCTTTCCCTACACGACGCTCTTCCGATCT) and P7 (GTGACTGGAGTTCAGACGTGTGCTCTTCCGATC) as the forward and reverse primers, respectively. In the first replicate, the previously reported primers, consisting of 12-, 10- and 15-primer sets for apple, pear and tea, respectively, were used in a single multiplex reaction. For the second replicate of this experiment, we divided each primer set into 2 subsets, i.e., 6 primers × 2 for apple, 5 primers × 2 for pear and 7 and 8 primers for tea. PCR amplification was performed in a 10-µL reaction containing 5 µL QIAGEN Multiplex PCR Kit (QIAGEN), 0.2 µL primer solution (10 μM each of forward and reverse), 3.8 µL H_2_O, and 1 µL genomic DNA. The PCR was performed using the following steps: an initial denaturation at 95 °C for 15 min followed by 25 cycles of denaturation at 95 °C for 30 s, primer annealing at 55 °C for 1 min, and extension at 72 °C for 30 s and, finally, followed by a final extension at 72 °C of 10 min. The second PCR was performed using long primers P5 (AATGATACGGCGACCACCGAGATCTACAC[Index]ACACTCTTTCCCTACACGACG) and P7 (CAAGCAGAAGACGGCATACGAGAT[Index]GTGACTGGAGTTCAGACGTGT) in which Illumina flow-cell binding sites and two different indexes of 8 bp were contained. The primers used for this experiment are listed in Table S1. PCR amplification was performed in a 10-µL total volume consisting of 5 µL 2× Green GoTaq G2 Hot Start Master Mix (0.4 mM each dNTP, Taq DNA polymerase and 4 mM MgCl_2_, pH 8.5; Promega), 1 µL P5 and P7 primers (1 μM) 3 µL H_2_O and 1 µL of the first PCR products. PCR reactions included the following steps: an initial denaturation of 95 °C for 5 min followed by 15 cycles of 94 °C for 30 s, 60 °C for 1 min and 72 °C for 30 s. This was followed by a final extension of 10 min. All of the second PCR products were mixed equally in a single tube and purified using AMPure XP beads (Beckman Coulter, Inc., Bree, CA, USA) following the Agencourt AMPure XP PCR Purification protocol. The library was sequenced using PE 300-bp sequencing on an Illumina MiSeq platform (Illumina, Inc., San Diego, CA, USA).

The reads from Illumina MiSeq were demultiplexed to each variety based on the index sequences using the Illumina system, and the paired fastq files of each variety were obtained. We used flash2 [45] to merge the paired fastq files with parameter “-M 300 -× 0.5 —allow-outies” (described in the script ‘“1.combine.sh”). The merged reads were then demultiplexed based on primer sequences by using the script “2.demultiplex.sh”. The number of reads of each allele was counted for each variety by using the script “3.CountSSR.sh”. (These scripts are provided in Files S1, S2 and S3, respectively.) We took only the four most common alleles, and the allele frequencies of the four were calculated. Only the first and second alleles were used to determine the genotypes of varieties, but the third and fourth alleles helped in detecting the presence of a stutter band and the accuracy of the called genotype.

To construct a database useful for both SSR-GBS and PCR-CE, we thoroughly compared the genotypes obtained by SSR-GBS with those obtained by PCR-CE and vice versa. First, the markers that showed fewer than 50 average reads in SSR-GBS were discarded. The correspondence of each allele of each marker obtained by SSR-GBS to the alleles obtained by PCR-CE was confirmed manually to identify cases of allele dropping in SSR-GBS. If allele dropping was found with a marker, that marker was not included in the database for SSR-GBS.

### 4.4. Automated Genotyping in SSR-GBS and Repeatability

In previous studies of SSR-GBS, allele calling was performed based on whether the frequency of the first allele exceeded 0.9 or on manual evaluation [27]. However, each marker has different features such as stutter bands and base variations in the primer binding site, making it difficult to set a single value for all marker sets. Here we set the variable α, which was determined separately for each marker. If the frequency of the first (most common) allele exceeded α, we called the genotype a homozygote for the first allele; otherwise, we called it a heterozygote for the first and second alleles (described in the script ‘4.genotyping.sh’ provided in File S4). The range of α for each marker was determined based on the criterion that the genotypes identified by SSR-GBS and PCR-CE were exactly the same for each of the varieties. In other words, the α values were set so that the genotyping results obtained by SSR-GBS in an automated system would match those previously obtained using PCR-CE, in which genotyping was done by eye. As an example of file structure, the files used to designate the apple cultivars and primers in this pipeline are provided in Files S5 and S6, respectively.

## Figures and Tables

**Table 1:** ID numbers, variety names and accession numbers for the 116 varieties used in this study, including 43 apple, 29 pear and 44 tea varieties.

Apple			Pear			Tea		
ID	Variety	Accession no. ^a^	ID	Variety	Accession no. ^a^	ID	Variety	Accession no. ^a^
APPLE01	Akane	JP169082	PEAR01	Akiakari	JP118536	TEA01	Ryofu	NA
APPLE02	Akibae	NA	PEAR02	Akizuki	JP118538	TEA02	Cha Chukanbohon No 3	NA
APPLE03	Iwakami	JP114453	PEAR03	Atago	JP113570	TEA03	Harumidori	NA
APPLE04	Indo	JP169681	PEAR04	Amanogawa	JP113562	TEA04	Sofu	NA
APPLE05	Orin	JP172644	PEAR05	Oushuu	JP118539	TEA05	Cha Chukanbohon No 4	JP232165
APPLE06	Ozenokurenai	NA	PEAR06	Okusankichi	JP113634	TEA06	Cha Chukanbohon No 5	JP232219
APPLE07	Gala	JP114085	PEAR07	Natsushizuku	JP230439	TEA07	Cha Chukanbohon No 6	NA
APPLE08	Kio	NA	PEAR08	Kumoi	JP113623	TEA08	Sun Rouge	NA
APPLE09	Kizashi	JP114525	PEAR09	Kousui	JP113619	TEA09	Shuntaro	NA
APPLE10	Kitakami	JP114148	PEAR10	Shuugyoku	JP113707	TEA10	Saeakari	NA
APPLE11	Kitaro	NA	PEAR11	Shuurei	JP118537	TEA11	Nanmei	NA
APPLE12	Kinsei	JP172634	PEAR12	Shinkou	JP113657	TEA12	Seimei	NA
APPLE13	Jonathan	JP169679	PEAR13	Shinsui	JP113660	TEA13	Sayamakaori	NA
APPLE14	Kotaro	NA	PEAR14	Shinsei	JP113694	TEA14	Yabukita	JP168695
APPLE15	Golden Delicious	JP116722	PEAR15	Suisei	JP113665	TEA15	Asatsuyu	NA
APPLE16	Sansa	JP114526	PEAR16	Chikusui	JP113716	TEA16	Okumidori	NA
APPLE17	Santaro	NA	PEAR17	Choujuurou	JP113574	TEA17	Kanayamidori	NA
APPLE18	Shinano Sweet	NA	PEAR18	Niitaka	JP113630	TEA18	Saemidori	JP171014
APPLE19	Jonagold	JP172630	PEAR19	Nijisseiki	JP113631	TEA19	Yutakamidori	JP168749
APPLE20	Sensyu	JP114288	PEAR20	Hakkou	JP113585	TEA20	Kiyoka	NA
APPLE21	Chinatsu	NA	PEAR21	Hayatama	JP113591	TEA21	MK5601	NA
APPLE22	Tsugaru	JP172651	PEAR22	Hougetsu	JP113720	TEA22	Kanaemaru	NA
APPLE23	Delicious	JP114041	PEAR23	Housui	JP113598	TEA23	Yachaken01	NA
APPLE24	Hatsuaki	JP169705	PEAR24	Yasato	JP113718	TEA24	Yachaken02	NA
APPLE25	Himekami	JP169714	PEAR25	Hatsumaru	NA	TEA25	Yachaken09	NA
APPLE26	Fuji	JP114078	PEAR26	Rinka	NA	TEA26	Yachaken10	NA
APPLE27	Hokuto	JP169715	PEAR27	Hoshiakari	NA	TEA27	Kokken01	NA
APPLE28	Maypole	NA	PEAR28	Narumi	NA	TEA28	Danshin37	NA
APPLE29	Mori-no-kagayaki	NA	PEAR29	Kanta	NA	TEA29	Miyazaki39	NA
APPLE30	Yoko	JP114416				TEA30	Miyazaki40	NA
APPLE31	Ruby Sweet	NA				TEA31	Okuharuka	NA
APPLE32	Rose Pearl	NA				TEA32	Kirari31	NA
APPLE33	JM1	NA				TEA33	Sainomidori	NA
APPLE34	JM2	NA				TEA34	Sakimidori	NA
APPLE35	JM5	NA				TEA35	Nagomiyutaka	NA
APPLE36	JM7	NA				TEA36	Haruto34	NA
APPLE37	JM8	NA				TEA37	Harunonagori	NA
APPLE38	Morioka 66	NA				TEA38	Harumoegi	NA
APPLE39	Beniminori	NA				TEA39	Miyamakaori	NA
APPLE40	Morioka 68	NA				TEA40	Musashikaori	NA
APPLE41	Morioka 69	NA				TEA41	Yumekaori	NA
APPLE42	Kinshu	NA				TEA42	Yumewakaba	NA
APPLE43	Morioka 71	NA				TEA43	Benifuki	NA
						TEA44	Sayamaakari	NA

^a^ Accession no. for NARO Genebank (www.gene.affrc.go.jp). NA means that the variety is not registered in NARO Genebank.

**Table 2:** Number of alleles, observed and expected heterozygosity and polymorphism information content of the SSR loci determined by PCR-CE.

Marker	Plant	No. of Alleles	*H*_O_	*H*_E_	PIC
CH01b09b ^a^	Apple	3	0.59	0.67	0.52
Hi08h08	Apple	3	0.56	0.54	0.47
Hi09f01	Apple	4	0.79	0.63	0.57
Hi15h12	Apple	3	0.26	0.29	0.26
Hi22d06	Apple	5	0.79	0.74	0.69
Mdo.chr1.18	Apple	5	0.63	0.64	0.57
NZmsCN943067	Apple	5	0.61	0.51	0.46
NZmsEB116209	Apple	3	0.70	0.66	0.58
NzmsEB119405 ^a^	Apple	4	0.52	0.61	0.48
NzmsEB146613	Apple	3	0.74	0.63	0.56
SamsCN944528	Apple	5	0.65	0.61	0.55
SamsEB132187	Apple	3	0.65	0.60	0.52
Averages for apple markers		3.8	0.62	0.59	0.52
TsuGNH124	Pear	2	0.55	0.49	0.37
TsuGNH161	Pear	3	0.62	0.60	0.52
TsuGNH164 ^a^	Pear	3	0.57	0.41	0.49
TsuGNH179	Pear	3	0.83	0.59	0.52
TsuGNH184 ^a^	Pear	2	0.50	0.48	0.37
TsuGNH194	Pear	2	0.35	0.37	0.30
TsuGNH204	Pear	2	0.03	0.03	0.03
TsuGNH207	Pear	2	0.28	0.24	0.21
TsuGNH208	Pear	3	0.45	0.52	0.46
TsuGNH250	Pear	2	0.41	0.33	0.27
Averages for pear markers		2.4	0.46	0.41	0.35
CsFM1097	Tea	4	0.43	0.49	0.45
CsFM1206	Tea	3	0.50	0.57	0.50
CsFM1566	Tea	4	0.61	0.49	0.45
CsFM1595 ^a^	Tea	5	0.62	0.63	0.57
MSE0348	Tea	5	0.71	0.61	0.56
MSE0354 ^a^	Tea	4	0.64	0.80	0.57
TM043 ^a^	Tea	5	0.45	0.46	0.40
TM107	Tea	6	0.80	0.69	0.64
TM336 ^a^	Tea	3	0.35	0.30	0.31
TM348	Tea	5	0.64	0.59	0.51
TM350 ^a^	Tea	4	0.36	0.36	0.33
TM464 ^a^	Tea	7	0.51	0.57	0.45
TM485	Tea	3	0.39	0.35	0.30
TM553	Tea	3	0.80	0.50	0.39
TM626	Tea	4	0.55	0.46	0.41
Averages for tea markers		4.3	0.56	0.52	0.46

*H*_O_ = Observed heterozygosity, *H*_E_ = Expected heterozygosity, PIC = Polymorphism information content, ^a^ This marker was not suitable for SSR-GBS because of the presence of allele dropout.

**Table 3:** Summary of each marker tested for SSR-GBS. Average depth indicates the number of the reads obtained after demultiplexing based on forward and reverse primer sequences in each marker. Replicated analyses with a slight change in the multiplex PCR set were conducted for validation.

Marker	Plant	Average Depth (Number of Reads)	Average Depth in Replicate Analysis	Difference in Size from the Alleles in PCR-CE (bp)	Result in SSR-GBS
CH01b09b	Apple	6834	5724	1	Errors due to stutter band in ‘Jonathan’ and ‘Sensyu’
Hi08h08	Apple	342	470	1	Comparable to the result of PCR-CE
Hi09F01	Apple	395	605	−4	Comparable to the result of PCR-CE
Hi15h12	Apple	5329	4331	0	Comparable to the result of PCR-CE
Hi22d06	Apple	3611	4070	3	Comparable to the result of PCR-CE
Mdo.chr1.18	Apple	1206	1108	−2	Comparable to the result of PCR-CE
NZmsCN943067	Apple	1777	994	−3	Comparable to the result of PCR-CE
NZmsEB116209	Apple	747	534	3	Comparable to the result of PCR-CE
NZmsEB119405	Apple	2442	2481	1	Allele dropout was observed in ‘JM1’, ‘JM2’, ‘JM5’, ‘JM8’
NZmsEB146613	Apple	8023	4636	1	Comparable to the result of PCR-CE
SAmsCN944528	Apple	6249	4003	−1	Comparable to the result of PCR-CE
SAmsEB132187	Apple	6621	4034	−3	Comparable to the result of PCR-CE
TsuGNH124	Pear	75	760	−2	Comparable to the result of PCR-CE
TsuGNH161	Pear	16,758	23,807	2	Comparable to the result of PCR-CE
TsuGNH164	Pear	716	1682	−3	The read depths were too low to determine genotype in ‘Oushuu’
TsuGNH179	Pear	1671	4622	−3	Comparable to the result of PCR-CE
TsuGNH184	Pear	21	116	−1	The read depths were too low to determine genotypes
TsuGNH194	Pear	25,406	12,843	1	Comparable to the result of PCR-CE
TsuGNH204	Pear	3877	1957	−2	Comparable to the result of PCR-CE
TsuGNH207	Pear	2718	1231	2	Comparable to the result of PCR-CE
TsuGNH208	Pear	7348	3735	−1	Comparable to the result of PCR-CE
TsuGNH250	Pear	5021	2847	−2	Comparable to the result of PCR-CE
CsFM1097	Tea	1217	1868	−4	Comparable to the result of PCR-CE
CsFM1206	Tea	1259	1396	−2	Comparable to the result of PCR-CE
CsFM1566	Tea	857	1174	−1	Comparable to the result of PCR-CE
CsFM1595	Tea	195	268	0	The read depths were too low to determine genotype in ‘Cha Chukanbohon No. 6’
MSE0348	Tea	3580	2559	0	Comparable to the result of PCR-CE
MSE0354	Tea	10,510	8783	1	Allele dropout was observed in some varieties
TM043	Tea	3469	2528	0	Allele dropout was observed in some varieties
TM107	Tea	2249	1645	−3	Comparable to the result of PCR-CE
TM336	Tea	14	12	1	The read depths were too low to determine genotypes
TM348	Tea	691	577	−2	Comparable to the result of PCR-CE
TM350	Tea	514	357	−3	Allele dropout was observed in some varieties
TM464	Tea	546	371	0	Allele dropout was observed in some varieties
TM485	Tea	2365	2947	−2	Comparable to the result of PCR-CE
TM553	Tea	6800	8285	0	Comparable to the result of PCR-CE
TM626	Tea	1605	2288	0	Comparable to the result of PCR-CE

**Table 4:** Optimum range of variable α and correlation of first-allele frequency between the two replicates for each marker. The range of α for each marker was determined based on the criterion that the genotypes identified by SSR-GBS and PCR-CE were exactly the same for each of the varieties.

Marker	Plant	Minimum Value of α	Optimum Value of α	Maximum Value of α	Correlation of First-Allele Frequency between Two Replicates
Hi08h08	Apple	0.45	0.57	0.69	0.98
Hi09F01	Apple	0.69	0.80	0.90	0.98
Hi15h12	Apple	0.34	0.43	0.52	0.98
Hi22d06	Apple	0.57	0.74	0.90	1.00
Mdo.chr1.18	Apple	0.60	0.61	0.61	0.98
NZmsCN943067	Apple	0.57	0.66	0.74	0.99
NZmsEB116209	Apple	0.55	0.70	0.84	0.99
NZmsEB146613	Apple	0.58	0.73	0.87	1.00
SAmsCN944528	Apple	0.64	0.78	0.91	0.99
SAmsEB132187	Apple	0.76	0.82	0.88	1.00
TsuGNH124	Pear	0.52	0.60	0.67	0.97
TsuGNH161	Pear	0.53	0.73	0.93	1.00
TsuGNH179	Pear	0.89	0.93	0.97	0.99
TsuGNH194	Pear	0.53	0.75	0.96	1.00
TsuGNH204	Pear	0.43	0.62	0.80	0.99
TsuGNH207	Pear	0.53	0.75	0.97	1.00
TsuGNH208	Pear	0.87	0.89	0.91	0.99
TsuGNH250	Pear	0.53	0.75	0.97	1.00
CsFM1097	Tea	0.55	0.71	0.87	0.99
CsFM1206	Tea	0.63	0.77	0.90	1.00
CsFM1566	Tea	0.71	0.83	0.95	0.99
MSE0348	Tea	0.73	0.85	0.97	0.99
TM107	Tea	0.72	0.85	0.98	0.98
TM348	Tea	0.61	0.77	0.93	0.99
TM485	Tea	0.72	0.84	0.96	1.00
TM553	Tea	0.87	0.92	0.97	1.00
TM626	Tea	0.73	0.85	0.97	1.00

## Data Availability

The genotype data and the scripts used in this study are available in Supplementary files.

## Supplementary Materials

The following are available online at https://www.mdpi.com/article/10.3390/plants10122796/s1, Table S1: SSR markers used in the present study, Table S2: Genotypes determined by PCR-CE for 43 apple varieties, Table S3: Genotypes determined by PCR-CE for 29 pear varieties, Table S4: Genotypes determined by PCR-CE for 44 tea varieties, Table S5: Genotypes determined by SSR-GBS for 43 apple varieties, Table S6: Genotypes determined by SSR-GBS for 29 pear varieties, Table S7: Genotypes determined by SSR-GBS for 44 tea varieties, Figure S1: UPGMA dendrograms showing clustering based on simple allele sharing distance among (A) 44 apple, (B) 29 pear and (C) 44 tea varieties. The figures in red indicate an approximately unbiased (AU) *p* value that represents more accurate values of bootstrap probability. File S1: 1.combine.sh. Bash script for combining the pairs of fastq files, File S2: 2.demultiplex.sh. Bash script for demultiplexing based on primer sequences, File S3: 3.CountSSR.sh. Bash script for counting alleles detected in each variety, File S4: 4.genotyping.sh. Bash script for determining the genotypes automatically based on variable α, File S5: list_geno. List of apple varieties or IDs used in this study, File S6: list_primer. List of apple primers and variable α used in this study.
