# Supplementary material for: Development of SSR Databases Available for Both NGS and Capillary Electrophoresis in Apple, Pear and Tea
Source: Plants (Basel). 2021 Dec 17;10(12):2796. doi: 10.3390/plants10122796 (PMC8703814; doi:10.3390/plants10122796)
Supplement: Supplementary file 1 [file plants-10-02796-s001.zip › SUP/SupplementaryFig.pptx]

## Slide 1
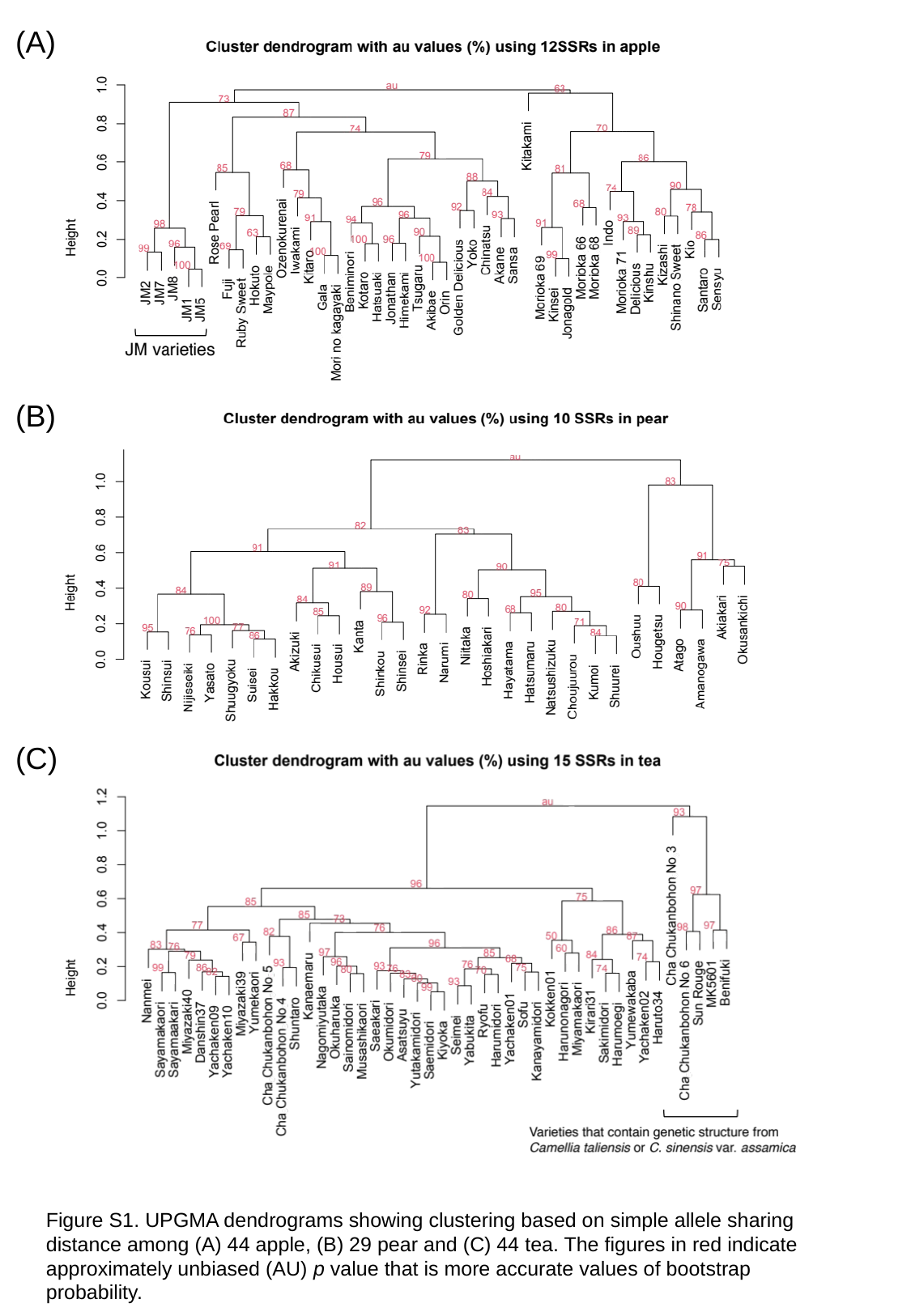

(A)
(B)
(C)
Figure S1. UPGMA dendrograms showing clustering based on simple allele sharing distance among (A) 44 apple, (B) 29 pear and (C) 44 tea. The figures in red indicate approximately unbiased (AU) p value that is more accurate values of bootstrap probability.
